# The precise determination of the window of implantation significantly improves ART outcomes

**DOI:** 10.1038/s41598-021-92955-w

**Published:** 2021-06-28

**Authors:** M. Enciso, J. Aizpurua, B. Rodríguez-Estrada, I. Jurado, M. Ferrández-Rives, E. Rodríguez, E. Pérez-Larrea, A. B. Climent, K. Marron, J. Sarasa

**Affiliations:** 1 IGLS Alicante, C/Britania 7, Alicante, Spain; 2 IVF Spain, Av. Ansaldo 13, 03540 Alicante, Spain; 3 IVF Donostia, Av. Tolosa 71-73, 20018 San Sebastian, Spain; 4 Sims IVF Clinic, Clonskeagh Road, Clonskeagh, Dublin, Ireland

**Keywords:** Genetics, Medical research, Molecular medicine

## Abstract

The human endometrium is receptive to the embryo for a specific period of time known as the window of implantation (WOI). During this period, the endometrium shows a specific gene expression profile suitable for endometrial function evaluation. ER Map is a molecular tool able to accurately predict endometrial receptivity status by transcriptomic analysis. In this retrospective study, including 2256 subfertile patients undergoing ART treatment, the clinical value of precise WOI determination is studied in detail. Results obtained when single embryo transfers (sET) were scheduled either within the WOI timeframe as established by ER Map, or deviating from this WOI, are assessed and compared. Data obtained showed that 34.18% (771/2256) of patients had a displaced WOI. Analysis of ART outcomes showed significantly higher pregnancy rates in transfers scheduled within the WOI predicted compared to transfers that deviated more than 12h from this WOI (44.35% vs 23.08%, p < 0.001). The deviation from the WOI had also an impact on the progression of pregnancy, with a significant increase in pregnancy loss (~ twofold) observed in transfers that deviated more than 12h from the WOI predicted. These results indicate that the precise determination of the WOI and personalised embryo transfer can significantly improve clinical outcomes.

## Introduction

One of the key processes for the establishment of a successful pregnancy is embryonic implantation into the endometrium, the mucous tissue that lines the inside of the uterus. For successful embryonic implantation, two essential elements are required: a competent embryo and an endometrium ready to receive it. Implantation involves an intricate dialog between the embryo and the endometrial matrix^1^. This interaction is essential for the apposition, adhesion and invasion of the blastocyst in the human endometrium^2^. Most ART developments in the last few years have focussed on, for the most part, the identification and selection of the most competent embryo to be transferred to the uterus. Less attention, however, has been paid to the other part of the story: the human endometrium. Very successful advanced embryo selection tools such as PGT-A or time lapse are now available and have rendered considerable improvements in ART results^3,4^, but still, only one side of the coin is being assessed when using these tools. Uterine ability to receive the embryo is not routinely given the same relevance.

During the menstrual cycle, the human endometrium undergoes important structural and functional changes in order to reach a status optimum for embryonic implantation. This period of time where the endometrium is receptive to the embryo is called the window of implantation (WOI) and it usually occurs between days 19 and 21 of the cycle^5,6^. In any other phase of the menstrual cycle, the endometrium is recalcitrant towards pregnancy^7^. It has been proposed that endometrial receptivity alterations may be the cause of implantation failure cases and may also be related with some early miscarriage cases^8^. Successful implantation requires both a viable embryo and synchrony between it and the receptive endometrium^9^. The precise identification and prediction of the period of uterine receptivity is key to improve the effectiveness of assisted reproduction treatments.

Recently, ER Map, a molecular tool for human endometrial receptivity evaluation based on the transcriptomic analysis of genes related to endometrial proliferation and embryonic implantation has been developed. ER Map uses a high-throughput RT-qPCR platform for the accurate evaluation of gene expression in endometrial samples. RT-qPCR has been shown to be the most accurate and reliable technique for gene expression analysis. ER Map tool allows the determination of transcriptomic profiles specifically associated to different endometrial receptivity status and accurate identification of the WOI^10^.

In the present study, we present results from the application of ER Map in the clinical setting in order to assess the efficacy of this tool for the precise determination of the WOI, the identification of cases of WOI displacement and the improvement of clinical outcomes following embryo transfer according to ER Map recommendation.

## Results

### Distribution of endometrial receptivity status in the patient population studied as predicted by ER Map

Endometrial receptivity status evaluation at the expected WOI timeframe by means of ER Map showed that 771 out of the 2256 patients evaluated (34.2%) had a displaced WOI. Within this group of NOT-RECEPTIVE patients, 25.0%, presented a pre-receptive endometrium, (i.e. had not reached the WOI yet) and 9.2% of patients a post-receptive uterine lining (i.e. had already passed their WOI).

With regards to age, no significant differences were detected in the frequencies of receptive and not receptive endometria found at P_4_ + 5.5 in HRT cycles as diagnosed by ER Map (X^2^ test, p > 0.05). No differences in age groups were found either in the frequencies of pre and post receptive results within the not receptive endometria (data not shown).

**Figure 1:**
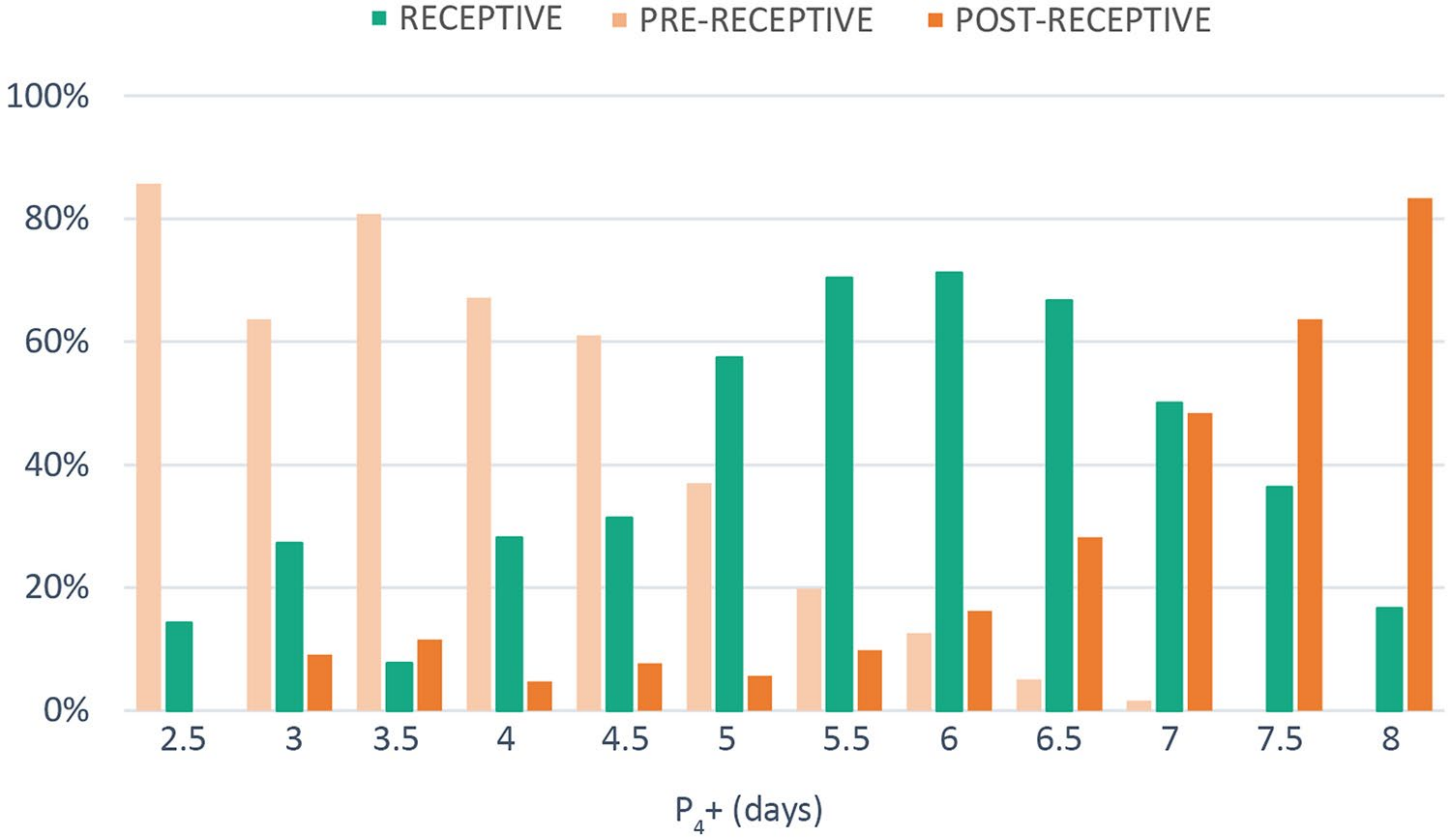
Endometrial receptivity status frequencies along different days of progesterone administration as assessed by ER Map. Frequencies of receptive, pre-receptive and post-receptive samples after a variable number of days of progesterone administration. N = 2828 biopsies.

The analysis of endometrial biopsies performed after a variable number of days of progesterone administration showed that patients´ endometria achieved receptive status within a wide variability of timeframes (P4 + 2.5 to P4 + 8) (Fig. 1). Most patients are receptive between 5 and 6 days of progesterone intake, however, we can find patients receptive as early as 2.5 days of P4 administration and even after 8 days of P4 intake.

### Reproducibility of ER Map results

The reproducibility of ER Map results between cycles of the same patient was also evaluated. The stability of the endometrial receptivity status within the same patient was explored in 29 women biopsied in two independent HRT cycles after the same progesterone pretreatment protocol. Characteristics of the biopsies and patients included in this analysis are shown in Supplementary Table 1. A 100% match of ER Map results (29 out of 29) was found in the group of biopsies analysed, confirming the existence of a specific timing of the WOI per individual patient and its stability between cycles (Supplementary Table 2).

### Clinical outcomes following ER Map testing

**Figure 2:**
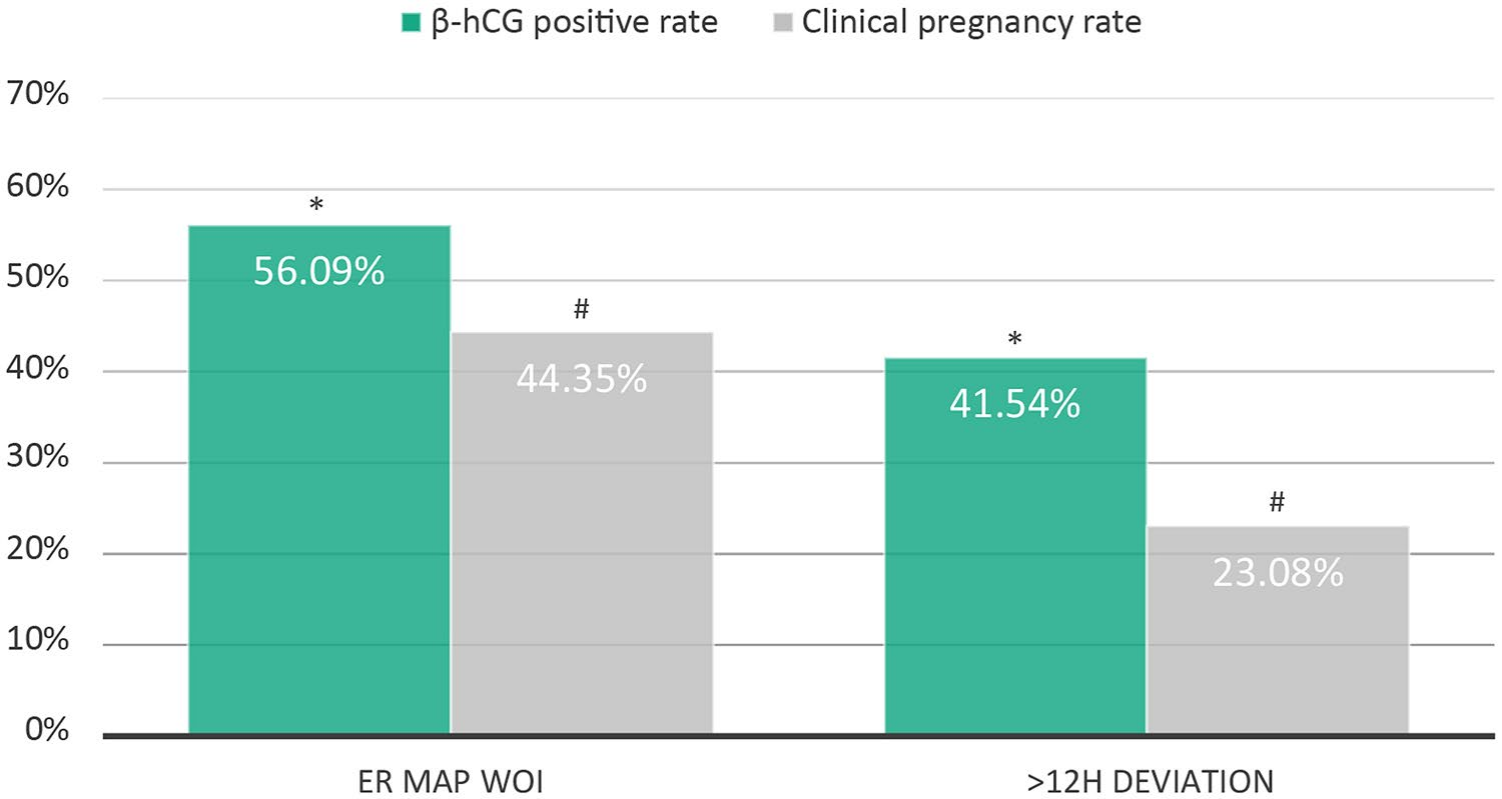
Reproductive outcomes of patients after the first single embryo transfer following ER Map test. Positive B-hCG rates (green bars) and clinical pregnancy rates (grey bars) comparison between the group of patients that followed ER Map progesterone pretreatment and those that deviated more than 12h from this recommendation. X^2^ test, *p = 0.02, ^#^p < 0.001 n = 746 sET (ER Map WOI N = 681, > 12h deviation n = 65).

Demographic and clinical characteristics of the patent population study groups are presented in Table 1. Analysis of outcomes after the first embryo transfer following ER Map test indicated a significantly higher positive β-hCG and clinical pregnancy rates (X^2^ test p = 0.02 and p < 0.001, respectively) in sETs scheduled within the WOI predicted by ER Map compared to transfers that deviated more than 12h from ER Map recommendation (Fig. 2). The deviation of embryo transfers from the WOI predicted by ER Map had also an impact on the progression of pregnancy. A significant increase in pregnancy loss (~ twofold) was detected in the group of transfers that deviated from ER Map recommendation compared to transfers performed within the ER Map WOI (PLR 44.44% vs 20.94%, X^2^ Test p = 0.005).

**Table 1:** Demographic and clinical characteristics of the patient population study groups.

	ER Map WOI	> 12 h deviation	p value
Sample size	681	65	–
Age (mean ± SD)	41.23 ± 4.74	41.15 ± 4.78	NS
**Type of cycle**			
Oocyte donation	517 (75.92%)	49 (75.38%)	NS
Autologous oocytes	164 (24.08%)	16 (24.62%)	NS
**Embryo development day at transfer**			
Day 3	68 (9.99%)	8 (12.31%)	NS
Day 5	613 (89.01%)	57 (87.69%)	NS
PGT-A cases	326 (47.90%)	23 (35.40%)	NS
Number of transferred embryos	1	1	–
Hours of exogenous P_4_ administration (mean ± SD)	133.57 ± 16.61	129.32 ± 25.12	0.034
Hours between ER Map WOI and ET (mean ± SD)	± 2.79 ± 4.8	± 30.83 ± 11.61	< 0.001

With the aim of analysing further the effect of the deviation from the WOI timeframe established by ER Map, clinical pregnancy rates of the group of transfers performed with a deviation of more than 24h were studied. Results showed a significant decline in pregnancy rates in transfers performed with a deviation from the WOI identified by ER Map of 24h or more (βR 56.09% vs 26.92%, X^2^ test p = 0.003; CPR 44.35% vs 19.23%; X^2^ test p = 0.011).

## Discussion

Results from the present study indicate that the analysis of endometrial gene expression signature by means of ER Map constitutes a valuable tool for the accurate identification of the WOI and thereby the improvement of ART results. Importantly, this study reveals the existence of a significant proportion of IVF patients with a “displaced” window of implantation and underlines the significance of precise embryo transfer synchronization to achieve a successful pregnancy.

Determination of the timing of endometrial receptivity and individualised preparation for embryo transfer seems to be key to improve implantation attainment and reduce miscarriage. Within the group of patients analysed, more than 30% showed a displaced WOI. This percentage matches the frequency found by other authors that determined receptivity status using molecular clustering methods^11–13^. In the tested group, WOI displacement was very variable, ranging from 12 to 60h. We detected patients with receptive endometria as early as 2.5 days after the first progesterone intake (P_4_ + 2.5) and up to 8 days after progesterone administration (P_4_ + 8). Such a wide variability of timeframes for the acquisition of endometrial receptivity may have important implications in the success of the treatment of many couples.

Several studies have shown that the endometrium is recalcitrant to pregnancy outside of the WOI^8,14^. However, no other study had previously defined the range of possible WOI timeframes nor highlighted the importance of a deviation of mere hours in the transference of the embryo and successful implantation^15^.

In our studies we have observed poor performance of embryo transfers programmed outside the WOI, with a considerable decline in pregnancy rates resulting from deviations of just 12h and a sharper decline when the transfers are performed with larger deviations (> 24h). This is a very interesting finding revealing that in this cohort of patients the precise determination of the WOI seems to be crucial for the achievement of good results.

A 2020 study on the impact of progesterone pretreatment times before transfer in egg donation cycles and frozen embryo transfers has reported similar results in protocols with 48–72h duration difference^16^. However, there is evidence of higher biochemical pregnancy rates and lower implantation rates when P4 supplementation begins earlier^17,18^. Many experiences on personalisation of progesterone pre-treatment before transfer have described improved results with synchronisation of embryo transfer and the window of implantation^19,20^. In spite of these findings, however, the embryo transfer strategy applied by most centres worldwide does not take into account precise WOI determination. Most clinics transfer blastocysts after 5 days of progesterone intake (with a range from P_4_ + 4 to P_4_ + 6) and they normally use unique protocols for all patients, usually no personalisation of endometrial preparation is performed. If we take into account that the majority of embryo transfers are performed after 5 days of P_4_ administration, in the population analysed, this means that more than one third of transfers would be done with greater than a 12-h difference relative to the WOI, and hence, will significantly compromise treatment success (doubling miscarriage rate and reducing implantation rate by approximately one fourth).

In light of our data, we believe individualisation of endometrial preparation before embryo transfer is crucial to guarantee the best diagnosis and maximise the efficacy of the treatment in not just the patient populations studied but also perhaps in broader patient groups. The personalised medicine approach is, in many areas widely acclaimed as being superior to results without personalised treatment^21,22^. The field of ART does and will benefit from further personalisation of treatment protocols. The identification of cases with alterations in endometrial receptivity in the first appointment can be useful for the adequate management of the infertile couple and the design of an efficient treatment strategy and IVF process. For many women the correct identification of the WOI may be the only option to achieve a pregnancy. Sadly, under current protocols this approach may not be recommended until several failures of good quality embryos have been experienced.

Although this is a retrospective study, to our knowledge, it compiles the largest dataset of clinical cases and outcomes following transcriptomic endometrial receptivity determination published so far. Moreover, unlike other studies, it includes a comparison group of patients that underwent endometrial biopsy and receptivity evaluation but no personalised transfer (group of transfers deviated from the WOI > 12h or > 24h) thus eliminating the possible bias of the so called “scratch effect”. The controversy around the clinical value of this practice is well known^23^. Some authors have suggested that the local injury produced by the scratch or endometrial biopsy may help embryo implantation in the subsequent month by way of wound release of cytokines and hormones^24^, while others have seen a detrimental or no effect of such injury on treatment success^25,26^. A recent RTC involving over a nine hundred women undergoing ART concludes no positive or negative effect of endometrial scratching^27^.

With regards to clinical outcomes, results from the application of ER Map in the clinical setting confirm that this tool significantly improves implantation and ongoing pregnancy rates. Personalisation of the progesterone pretransfer protocol and synchronised embryo transfer by means of ER Map achieves pregnancy rates of 56.09% after the first transfer of a single embryo in patients with previous failed IVF cycles. Other successful experiences of embryo transfer personalization using transcriptomic analyses have also reported improvements in ART outcomes achieving 57.3% implantation rates after the first pET^20^ and 44.6% beta positive rates per patient in customized transfers^19^. In the present study, deviation of embryo transfer from the WOI reduced pregnancy rates by one fourth and doubled pregnancy loss. In the group of transfers performed with a deviation of > 12h from the WOI, almost half of the cases that achieved a positive beta do not progress to 8 weeks. These results provide significant evidence regarding the importance of embryo transfer within the WOI not only for successful implantation but also for the progression of pregnancy. This idea had been already anticipated by some authors that suggested that implantation is possible in a broad window but only optimal in a narrower timeframe^28^. However, the impact of a 12h deviation from WOI on the probability of positive pregnancies to reach 8 weeks is here presented for the first time. This indicates that not only couples suffering from implantation failure but also couples that experience miscarriage could have a displaced WOI and could benefit from the synchronization of embryo transfer with its WOI. This influence of a deviation of hours may be the reason why some studies of personalised embryo transfer (pET) detect an unexpectedly high number of clinical miscarriages^29^. In the recent RCT by ERA, intention to treat analysis revealed 3 times more clinical miscarriages in the pET group compared to the fresh embryo transfer group^20^. A precise schedule of embryo transfer calculated by the hour to synchronise the transfer with the WOI may guarantee the best results.

The process of pregnancy failure is not well understood. Several lines of evidence have pointed out that although it is a multifactorial phenomenon, the main players determining pregnancy success are the viability of the embryo and the endometrial environment^30^.

Our results suggest an important influence of the endometrium and its transcriptomic changes not only on pre and peri-implantation processes but also on post embryo implantation stages. Other authors using transcriptomic analyses of the endometrium have described specific sub-signatures linked to different stages of the progression of pregnancy including biochemical and ongoing pregnancy^31^ but so far, to our knowledge, no other group had showed the impact of a deviation of hours from the WOI on pregnancy loss. The survival of the embryo in the uterus after implantation may be influenced not only by the embryo developmental potential but also by the degree of endometrial receptivity of the uterus in or around the moment of embryo transfer. The role of the endometrium in pregnancy loss has historical roots but remains controversial^32^. Interesting data from transcriptomic, proteomic and epigenomics studies are regularly being produced with great potential for diagnosis and treatment^33–35^. These findings may change the paradigm, and consider the important role of the endometrium in pregnancy in phases beyond implantation.

Although this is a controversial topic, with some authors questioning the usefulness of endometrial evaluation and personalised schedule of embryo transfer^36,37^ and others supporting it^19,20^, we believe that the clinical assessment of the endometrium still remains to be considered as an important part of the investigation of couples with unexplained pregnancy loss. Many practitioners that support the value of endometrial evaluation have however, thus far constrained the benefits of the evaluation of endometrial receptivity to patients with repeated implantation failure. There is no doubt that this group of patients will benefit from the identification of an endometrial factor as the cause of their infertility, but, from our point of view, the precise determination of the WOI could benefit a larger group of patients, such as those who have suffered from recurrent miscarriages, or those patients with idiopathic infertility in whom the origin of their reproductive problem is unknown. Given the expense and emotional hardship of both IVF and failed pregnancies, many patients may therefore benefit from shorter time to pregnancy through early identification of a displaced WOI, additionally women with low ovarian response, in which the production of good quality embryos is difficult and where the transfer of an embryo outside the implantation window can seriously compromise their option of becoming pregnant and finally, patients in a PGT-A program with advanced maternal age, where euploid embryos are scarce. In short, we believe that the analysis of endometrial receptivity may have benefits in a far wider range of infertility conditions outside those proposed to date.

Finally, we also present data on the reproducibility of ER Map. Results from the double analysis of biopsies in 29 patients and the improved ART results obtained in cases where transfers performed within the WOI identified by ER Map endorse the high reproducibility of the test. This high reproducibility may be due to the technique used for transcriptomic analyses and the robust experimental design of the tool. ER Map uses high-throughput RT-qPCR for the analysis of a carefully selected panel of genes involved in the process of endometrial proliferation and embryo implantation^10^. The RT-qPCR is the most robust and reliable technique currently available for gene expression analysis, currently considered the gold standard. Alternative methodologies output such as microarray results and RNA-seq expression data, produced by other tests such as ERA need to be validated using RT-qPCR methods^38^.

The high reproducibility of ER Map warrants that the application of this tool for the personalised schedule of embryo transfer can be confidently performed. Proving the reproducibility of an endometrial receptivity test is essential to guarantee the reliability of the tool, especially in this type of test where the intervention relies on the assumption that the effect of the endometrial preparation cycle for biopsy is similar to the endometrial preparation cycle for transfer. This idea is also supported by other groups^39,40^.

In summary, this study provides strong evidence that ER Map endometrial receptivity evaluation can reliably identify the WOI. The application of ER Map for the identification of cases of WOI displacement and personalised embryo transfer scheduling is an effective strategy for improving ART outcomes. Personalisation of progesterone duration pre-treatment before transfer renders significantly improved ART results, increasing the likelihood of pregnancy and reducing the risk of miscarriage. Not only patients suffering from implantation failure but a wider range of patient conditions such as couples experiencing recurrent miscarriage can benefit from the accurate identification of the WOI.

This study presents very encouraging results about the influence of the endometrium and the potential of the detailed study of endometrial receptivity in the efficiency of the diagnosis and treatment of infertility. Nevertheless, in order to determine the true extent of the clinical benefits here described, other types of investigations, such as non-selection studies and randomized controlled trials, will also be necessary.

## Material and methods

### Study design

This is a retrospective multicentre study analysing 2256 patients undergoing endometrial receptivity assessment by ER Map (2828 biopsies) for a variety of reasons including, recurrent implantation failure, recurrent miscarriage and idiopathic infertility between March 2016 and September 2019. Clinical outcomes obtained when single embryo transfers were scheduled either on the moment of endometrial receptivity (WOI timeframe) as recommended by ER Map, or deviating from this recommendation were analysed and compared. Clinical outcome measures were: positive β-HCG rate [βR], clinical pregnancy rate [CPR] and pregnancy loss rate [PLR].

### Ethical approval

Ethical approval for the study was obtained from the Hospital General de Alicante Ethics Committee (CEI PI2017/80.1). All research was performed in accordance to the principles of the Declaration of Helsinki. Informed consent was obtained from all participants.

### Endometrial preparation and biopsy

Endometrial preparation was performed in a Hormone Replacement Therapy (HRT) cycle. Briefly, oral estradiol 6 mg daily (PROGYNOVA 2 mg/8 h) was administered from day 1 of menses. Transvaginal sonography (TVS) was used to assess the pattern and thickness of the endometrium approximately 14 days after menses. After confirmation of trilaminar endometrium > 6.5 mm and a serum progesterone level < 1 ng/mL (3.2 nmol/L), progesterone 800 mg daily (UTROGESTAN 200 mgx2/12 h) was administered. The initial day of progesterone administration was deemed “P + 0”, and biopsy was performed with a Pipelle catheter after five and a half days of progesterone administration (132 h, P + 5.5). Endometrial biopsies of approximately 30 mg were obtained from the uterine fundus using a Pipelle catheter (GYNETICS, Namont-Achel, Belgium) under sterile conditions. Tissue was then transferred to a tube containing 1.5 mL of an RNA preservation media (RNAlater, Merck, Darmstadt, Germany) and stored for a minimum of 4 h at 4 °C and then shipped for ER Map processing (iGLS, Alicante, Spain).

### Endometrial Receptivity Map (ER Map) analysis and embryo transfer recommendation

Endometrial biopsy samples were processed for RNA extraction and RT-qPCR expression analysis of ER Map genes, as described elsewhere^10^. Results from the test allow the diagnosis of the receptivity status of the endometrium at the time of the biopsy and the identification of the WOI.

**Figure 3:**
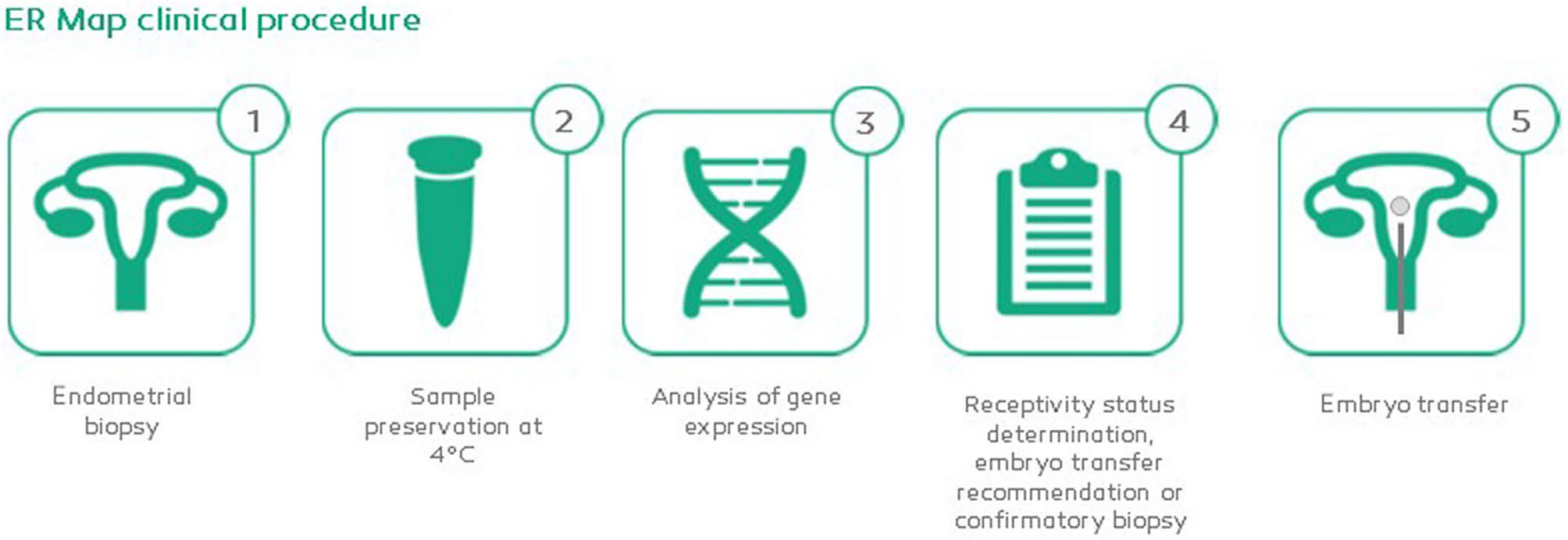
ER Map clinical procedure diagram. Modified from “[pking4th, alexutemov, greyjj] 123RF.com”.

Results from the test can classify an endometrium into: “receptive” or “not receptive” (either “pre-receptive” or “post receptive”). When an endometrium is classified as “receptive”, it means that the WOI matches the day on which the biopsy was taken. The embryo transfer is recommended to be scheduled on the same day and type of cycle on which the biopsy was taken. If an endometrium is classified as “not receptive”, either pre-receptive or post-receptive it means that the WOI is displaced. A second biopsy to confirm the displacement is recommended and embryo transfer scheduled accordingly (Fig. 3).

### Embryo culture, cryopreservation and transfer

All embryos were cultured to blastocyst stage and vitrified according to the standard protocols followed at each centre. The best quality embryos available were chosen for transfer. Single embryo transfers were performed in all cases. All transfers were performed in a subsequent cycle with frozen-thawed embryos under hormone replacement treatment cycle similar to the one where the endometrial biopsy was taken. Briefly, women received oestradiol (E2) priming. After confirmation of endometrial thickness > 6.5 mm and a serum progesterone level < 1 ng/mL (3.2 nmol/L), progesterone administration was started for a variable number of days, either following or deviating from ER Map recommendation.

On the day of the transfer, embryos were warmed and, 2 h post-thawing, assessed for survival and re-expansion. Embryo transfer was performed by vaginal ultrasound and soft catheter. The embryo was placed at 1 cm of uterine fundus in CSC medium previously drawn into the catheter.

### Data analysis and statistics

Frequencies of receptive, pre-receptive and post-receptive samples within the expected WOI (at P + 5.5) and after a variable number of days of progesterone administration were calculated.

The influence of age and endometrial receptivity status was evaluated by X^2^ test.

Clinical outcome measures including positive β-hCG rate [βR], clinical pregnancy rate [CPR] and pregnancy loss rate [PLR] between groups of transfers performed either on the WOI identified by ER Map or deviating from this recommendation were calculated and compared. Significant differences between clinical outcome values from the study groups were explored using X^2^ test.

Positive β-HCG rate [βR] was determined by a β-HCG positive result (β-hCG > 25 IU/L); Clinical pregnancy rate [CPR] was determined by ultrasonographic identification of an intrauterine gestational sac with foetal heartbeat at 8 weeks following embryo transfer. Pregnancy loss rate [PLR] was defined by the failure to detect by US 8 weeks after embryo transfer of an intrauterine gestational sac with foetal heartbeat following a positive β-HCG result.

Data analyses were performed by using IBM SPSS Statistics software version 19.0. A p-value of < 0.05 was considered to be statistically significant.

## Supplementary Information

Supplementary Table S1.

Supplementary Table S2.

## Data Availability

The datasets generated during and/or analysed during the current study are available from the corresponding author on reasonable request.
