# Supplementary material for: The precise determination of the window of implantation significantly improves ART outcomes
Source: Sci Rep. 2021 Jun 28;11:13420. doi: 10.1038/s41598-021-92955-w (PMC8238935; doi:10.1038/s41598-021-92955-w)
Supplement: Supplementary file 1 — Supplementary Table S1. [file 41598_2021_92955_MOESM1_ESM.docx]

**Supplementary Table I. Characteristics of patients biopsied and ER Map tested in two independent HRT cycles after the same progesterone pretreatment protocol.**

| Number of patients | 29 |
| --- | --- |
| Number of IVF Centres | 8 |
| Mean age 1st biopsy (years old) | 41.05 |
| Mean age 2nd biopsy (years old) | 42.01 |
| Biopsies performed at P4+ (range) | 4-7.5 |
| Number of Pre-receptive | 9 |
| Number of Receptive | 17 |
| Number of Post-receptive | 3 |
| Average time between biopsies (days) | 423 |
| Average time between biopsies Pre receptives (days) | 74 |
| Average time between biopsies Receptives (days) | 665 |
| Average time between biopsies Post receptives (days) | 93 |
