# Supplementary material for: The precise determination of the window of implantation significantly improves ART outcomes
Source: Sci Rep. 2021 Jun 28;11:13420. doi: 10.1038/s41598-021-92955-w (PMC8238935; doi:10.1038/s41598-021-92955-w)
Supplement: Supplementary file 2 — Supplementary Table S2. [file 41598_2021_92955_MOESM2_ESM.docx]

**Supplementary Table II. ER Map test results of the same patient biopsied in two independent HRT cycles after the same progesterone pretreatment protocol.** N=29.

| Patient | 1st biopsy  ER Map® result | Performed at P4+ | 2nd biopsy  ER Map® result | Performed at P4+ |
| --- | --- | --- | --- | --- |
| 1 | Post-receptive | 7 | Post-receptive | 7 |
| 2 | Receptive | 6.5 | Receptive | 6.5 |
| 3 | Receptive | 6 | Receptive | 6 |
| 4 | Receptive | 7 | Receptive | 7 |
| 5 | Receptive | 6 | Receptive | 6 |
| 6 | Receptive | 6 | Receptive | 6 |
| 7 | Receptive | 6 | Receptive | 6 |
| 8 | Pre-receptive | 5 | Pre-receptive | 5 |
| 9 | Receptive | 5.5 | Receptive | 5.5 |
| 10 | Receptive | 6 | Receptive | 6 |
| 11 | Post-receptive | 6.5 | Post-receptive | 6.5 |
| 12 | Pre-receptive | 5 | Pre-receptive | 5 |
| 13 | Receptive | 6 | Receptive | 6 |
| 14 | Post-receptive | 5 | Post-receptive | 5 |
| 15 | Receptive | 5.5 | Receptive | 5.5 |
| 16 | Pre-receptive | 4.5 | Pre-receptive | 4.5 |
| 17 | Receptive | 5.5 | Receptive | 5.5 |
| 18 | Pre-receptive | 5.5 | Pre-receptive | 5.5 |
| 19 | Receptive | 6 | Receptive | 6 |
| 20 | Pre-receptive | 5.5 | Pre-receptive | 5.5 |
| 21 | Receptive | 7.5 | Receptive | 7.5 |
| 22 | Pre-receptive | 4 | Pre-receptive | 4 |
| 23 | Receptive | 5.5 | Receptive | 5.5 |
| 24 | Pre-receptive | 5 | Pre-receptive | 5 |
| 25 | Receptive | 5.5 | Receptive | 5.5 |
| 26 | Receptive | 6 | Receptive | 6 |
| 27 | Pre-receptive | 5 | Pre-receptive | 5 |
| 28 | Pre-receptive | 5 | Pre-receptive | 5 |
| 29 | Receptive | 5.5 | Receptive | 5.5 |
